# Supplementary material for: Multipartite state generation in quantum networks with optimal scaling
Source: Sci Rep. 2019 Jan 22;9:314. doi: 10.1038/s41598-018-36543-5 (PMC6342956; doi:10.1038/s41598-018-36543-5)
Supplement: Supplementary file 1 — Supplementary Material [file 41598_2018_36543_MOESM1_ESM.pdf]

Supplementary Material
Multipartite state generation in quantum networks with optimal scaling

J. Wallnöfer, A. Pirker, M. Zwerger, and W. Dür

Institut für Theoretische Physik, Universität Innsbruck, Technikerstr. 21a, A-6020 Innsbruck, Austria

A Fidelity estimation for multipartite hashing with a finite number of input pairs

Continuing from the introduction in the main text, we detail how the fidelity estimate for the multipartite quantum hashing protocol is obtained. This estimate is especially relevant in the case that we consider here, namely applying the protocol with a limited number of input copies. Bennet's inequality to estimate the fidelity of the bipartite hashing protocol was already used in [1, 2]. While error estimates of the multipartite protocol were present in those publications, the details of the fidelity estimate is the new aspect regarding the multipartite quantum hashing protocol presented in this paper.

The global fidelity of the hashing protocol can be estimated by looking at the probability that the initial states fullfil the requirements assumed in the formulation of the protocol. While this gets exponentially more likely as the number of input copies n grows, we are interested in precisely the case where n is relatively small.

There are two error sources that might cause the hashing protocol to fail if n is kept finite, which in turn decreases the fidelity. First, the bitstring $\widetilde{a}$ might fall outside the likely subspace. We use Bennett's inequality to bound this probability, i.e. the probability that the sample entropy $\widetilde{S}(\widetilde{a})$ differing from the von Neumann entropy $S(\rho)$ by more than δ:

$$\mathrm{P}\left(\left|S(\rho)-\widetilde{S}(\widetilde{a})\right|>\delta\right)\leq 2\exp\left\{-n\frac{V}{a^2}h\left(\frac{a\delta}{V}\right)\right\} \tag{A.1}$$

with $a=\max_i|-\log_2\lambda_i-S(\rho)|$, $V=\sum_i\lambda_i\log_2^2\lambda_i-S^2(\rho)$ and $h(u)=(1+u)\log(1+u)$. For sufficiently high n this expression scales like $\alpha\exp\left(-\beta n\delta^2\right)$ for some α and β that depend on the input states.

Second, it might not be possible to uniquely identify $\widetilde{a}$ after a finite number of parity measurements, even if it falls into the likely subspace. The probability that any two different bitstrings show the same result after measuring a random subset parity is 1/2. From the noiseless coding theorem we know that the likely subspace contains at most $2^{n(S(\rho)+\delta)}$ states, the probability of not uniquely identifying $\widetilde{a}$ after k steps (consuming k input states) is smaller than $2^{n(S(\rho)+\delta)-k}$.

We choose $k=n(S(\rho)+2\delta)$, as suggested in [3], and obtain for the success probability, i.e. the global fidelity of all output states being in the desired state:

$$F\geq 1-2\exp\left\{-n\frac{V}{a^2}h\left(\frac{a\delta}{V}\right)\right\}-2^{-n\delta}=f(\widetilde{a},n,\delta) \tag{A.2}$$

Note that in the scenarios we consider one chooses the number of input qubits n and output qubits m and derives δ:

$$\delta=1/2\left(1-S(\rho)-m/n\right) \tag{A.3}$$

$\delta>0$ has to be met in order for the protocol to be viable with the given parameters, which also directly imposes a condition on possible values of m.

Now, as mentioned in the main part, for multipartite states a separate subprotocol is needed for each color of the graph and one bitstring per qubit has to be considered separately. The relevant entropy for the k-th qubit substring is given by S_k:

$$S_k=S(a^{(k)})=-\lambda_{k,0}\log_2\lambda_{k,0}-\lambda_{k,1}\log_2\lambda_{k,1} \tag{A.4}$$

where $\lambda_{k,i}=\sum_{\mu_{j\neq k}}\lambda_{\mu_1\ldots\mu_{k-1}i\mu_{k+1}\ldots\mu_N}$ is the probability that the k-th bit in the graph state basis vector $\boldsymbol{\mu}$ equals i.

For two-colorable graph states we define $S_A=\max_{k\in A}S_k$ and $S_B=\max_{k\in B}S_k$ for the vertices with colors A or B, respectively. Because $m/n=1-S_A-S_B-2\delta_A-2\delta_B$, only the sum of δ_A and δ_B is fixed, and we have to choose how to distribute these additional pairs between the subprotocols which can have an important effect for some error types (see section *Restricted error model* in the main text). The asymptotic yield is given by $m/n=1-S_A-S_B$ as δ_A and δ_B are allowed to approach 0 as n tends to infinity.

We define

$$f_k=1-2\exp\left\{-n\frac{V_k}{a_k^2}h\left(\frac{a_k\delta_k}{V_k}\right)\right\}-2^{-n\delta_k} \tag{A.5}$$

where $a_k=\max_i|-\log_2\lambda_{k,i}-S_k|$ and $V_k=-\lambda_{k,0}\log_2\lambda_{k,0}-\lambda_{k,1}\log_2\lambda_{k,1}$. If the noise is symmetric such that all S_k are equal for $k\in A$ then also $\delta_k=\delta_A$ have the same value. However, since all the bitstrings for one color can be evaluated simultaneously, a smaller S_k means that for that k δ_k is automatically chosen larger as even more copies are used. It holds that $\delta_k=\delta_A+(S_A-S_k)/2$ for $k\in A$. An anologous rule is derived for color B.

Finally, the global fidelity can be estimated by simply multiplying the success probabilites for the individual strings:

$$F\geq\prod_{k\in V}f_k \tag{A.6}$$

B Error model from connected Bell pairs

In the main text we discuss a model based on Bell pairs where one qubit has been affected by local depolarizing noise with error parameter q (as defined in equation (4) in the main text). These Bell pairs are then connected to a two-dimensional cluster state. In this section we derive the error pattern on that cluster state which arises from the noise on the Bell pairs.

One central property of the graph state basis is that any Pauli-diagonal noise channel can be written as a combination of correlated Z-noises (see e.g. [4]). We consider the graph state corresponding to the graph of two connected vertices 1 and 2 that can be written as $|G_{\mathrm{bip}}\rangle^{(1,2)} = 1/\sqrt{2}\left(|0\rangle_1 |+\rangle_2 + |1\rangle_1 |-\rangle_2\right)$, which clearly is local-Clifford equivalent to the standard Bell states. Local depolarizing noise acting on one qubit of that graph state can be described as:

$$q\mu + \frac{1-q}{3}\left(Z^{(1)}\mu Z^{(1)} + Z^{(2)}\mu Z^{(2)} + Z^{(1)}Z^{(2)}\mu Z^{(2)}Z^{(1)}\right) \tag{B.1}$$

where $\mu = |G_{\mathrm{bip}}\rangle\langle G_{\mathrm{bip}}|^{(1,2)}$.

Now we consider the situation where that state is connected to some other graph state $|G'\rangle$ with the connection procedure described in the main text (section *Connecting graph states*) using the qubit that we label 3 as the point where the bipartite graph state will be attached to $|G'\rangle$. The connection operation is given by $\mathrm{CNOT}^{2\to 3} = |0\rangle\langle 0|^{(2)} \otimes \mathbb{1}^{(3)} + |1\rangle\langle 1|^{(2)} \otimes X^{(3)}$ followed by a Z-measurement on qubit 3. Applying $\mathrm{CNOT}^{2\to 3}$ transforms the separate graph states to a new graph state corresponding to a graph where the new neighborhood N_2' of qubit 2 is given by $N_2 \bigcup N_3 - N_2 \bigcap N_3$.

First, we investigate what effect the noise on $|G_{\mathrm{bip}}\rangle$ has on the resulting state. $Z^{(2)}$ commutes with $\mathrm{CNOT}^{2\to 3}$ and also has no bearing on the outcome of the Z-measurement on qubit 3. Furthermore, with the possible local-Clifford corrections depending on the measurement outcome consist only of applications of Z to various qubits [4], which, again, commute with the noise pattern. Therefore, the noise pattern on the bipartite state is applied to the final graph state without any modifications.

Second, we also need to take into account that the state $|G'\rangle$ itself might be noisy. Again, any Pauli-diagonal noise on that state can be written as correlated Z-noises. Therefore it suffices to considers what happens if qubit 3 is affected by Z-noise. It holds that $\mathrm{CNOT}^{2\to 3}Z^{(3)} = Z^{(2)}Z^{(3)}\mathrm{CNOT}^{2\to 3}$. Therefore, $Z^{(3)}$ translates to $Z^{(2)}Z^{(3)}$ after the CNOT operation is applied. Again, the outcome of the Z-measurement and possible correction operations are not affected at all. So finally, every noise on the initial state $|G'\rangle$ that contained $Z^{(3)}$ translates to the same noise pattern with $Z^{(2)}$ on the final graph state.

Finally, applying these insights to our specific case gives rise to the noise pattern in equation (11) in the main text.

References

[1] Pirker, A., Zwerger, M., Dunjko, V., Briegel, H. J., and Dür, W. Simple proof of confidentiality for private quantum channels in noisy environments. arXiv:1711.08897 [quant-ph].

[2] Zwerger, M., Pirker, A., Dunjko, V., Briegel, H. J., and Dür, W. Long-Range Big Quantum-Data Transmission. *Phys. Rev. Lett. 120* (Jan 2018), 030503.

[3] Bennett, C. H., DiVincenzo, D. P., Smolin, J. A., and Wootters, W. K. "mixed-state entanglement and quantum error correction". *Phys. Rev. A 54* (Nov 1996), 3824–3851.

[4] Hein, M., Dür, W., Eisert, J., Raussendorf, R., van den Nest, M., and Briegel, H.-J. Entanglement in Graph States and its Applications. In *Quantum Computers, Algorithms and Chaos* (2006), vol. 162 of *Proceedings of the International School of Physics "Enrico Fermi"*, p. 115–218.
